# Association between Lymph Node Ratio and Disease Specific Survival in Breast Cancer Patients with One or Two Positive Lymph Nodes Stratified by Different Local Treatment Modalities

**DOI:** 10.1371/journal.pone.0138908

**Published:** 2015-10-29

**Authors:** Ruoxi Hong, Zhen Dai, Wenjie Zhu, Binghe Xu

**Affiliations:** 1 Department of Medical Oncology, Cancer Hospital, Chinese Academy of Medical Sciences and Peking Union Medical College, Beijing, China; 2 Chengdu Center for Disease Control and Prevention, Chengdu, China; School of Medicine, Fu Jen Catholic University, TAIWAN

## Abstract

**Purpose:**

Results of the American College of Surgeons Oncology Group (ACOSOG) Z0011 trial indicated that complete axillary node dissection (ALND) may not be warranted in some breast cancer patients with low tumor burden who are undergoing breast-conserving surgery following whole-breast irradiation. However, this study did not address patients undergoing mastectomy or those undergoing breast-conserving surgery without whole-breast radiotherapy. Given that lymph node ratio (LNR; ratio of positive lymph nodes to the total number removed) has been shown to be a prognostic factor in breast cancer, we first sought to determine the prognostic value of LNR in a low risk population comparable to that of the Z0011 trial and further to investigate whether the prognostic significance differs with local treatment modality.

**Method:**

We used the Surveillance Epidemiology and End Results (SEER) database to identify breast cancer patients with T1-T2 tumor and 1–2 positive nodes. Patients were subclassified by the local therapy they underwent for the primary tumor. The prognostic value of LNR in predicting disease-specific survival (DSS) was examined in each treatment group.

**Results:**

A total of 53,109 patients were included. In the subgroup of 20,602 patients who underwent lumpectomy following radiotherapy, LNR was not found to be significantly associated with DSS in both the univariate and multivariate model. For the 4,664 patients treated with mastectomy following radiotherapy, 6,811 treated with lumpectomy without radiotherapy and 21,031 with mastectomy without radiotherapy, LNR independently predict DSS in each of these subgroups.

**Conclusions:**

Our results *add evidence to the concept* that axillary dissection could be omitted in patients with one or two positive nodes following breast-conserving surgery and whole breast radiation.

## Introduction

Axillary lymph node dissection (ALND) has been an integral part of breast cancer surgery since the introduction of radical mastectomy by William Halsted [1]. ALND reliably identifies axillary lymph node metastases and results in a high rate of local control [2,3]. In the early 1990s, sentinel lymph node dissection (SLND) was developed to allow accurate staging of breast cancer patients as node-positive or node-negative with less morbidity than ALND [4]. Over the next decade, SLND gradually replaced ALND in assessing nodal status in clinical node-negative patients [5,6]. Therefore, the role of ALND is mostly limited to preventing local recurrence. Whether ALND confers a survival advantage is controversial [7–9].

Traditionally, ALND was mandatory for patients with positive SLNs. Data from the American College of Surgeons Oncology Group (ACOSOG) Z0011 trial has challenged the role of ALND in node-positive breast cancer [10,11]. This study observed no statistically significant difference in local recurrence and overall survival (OS) for SLND and ALND compared to SLND alone in patients with T1-T2 tumors and one or two positive SLNs who were treated with lumpectomy following radiotherapy. These results indicated that ALND could be safely omitted in select patients with a low axillary tumor burden. However, the Z0011 trial did not address patients undergoing mastectomy or those undergoing lumpectomy without radiotherapy. In those patients, the role of ALND is currently under debate when SLND identifies a positive SLN [12,13].

A number of authors have reported better prognosis when an adequate number of LNs was removed by ALND [14,15]. However all of these investigations are retrospective, and the number of nodes considered adequate for ALND is controversial [16,17]. In the past decade, a growing number of studies have introduced lymph node ratio (LNR), defined as the ratio of positive LNs to the total number of removed LNs, as an important prognostic factor [18–23]. A higher ratio would indicate a worse prognosis. Unfortunately, data regarding the prognostic value of LNR in patients with a low axillary tumor burden are limited.

The aim of the present study was to determine the prognostic value of LNR in a low risk population comparable to the Z0011 trial and to investigate whether the prognostic significance differs in patients receiving different local treatment modalities. We used the Surveillance Epidemiology and End Results (SEER) database to identify patients with T1-T2 tumor and one or two positive nodes. Patients were sub-classified by local therapy of the primary tumor, and the prognostic value of LNR was examined in each treatment group.

## Materials and Methods

### Study population

For this study, we used the SEER database November 2013 submission [24]. Female patients over the age of 18 who were diagnosed between 1998 and 2006 with T1-T2 and one or two nodes positive for primary invasive ductal carcinoma (IDC) or invasive lobular carcinoma (ILC) were reviewed. Patients were excluded if they had stage III or IV disease, did not undergo surgical resection, or if the total number of nodes examined was unknown.

Patients were categorized into 4 subgroups based on the local therapy they underwent: BCS+RT (lumpectomy following radiotherapy), BCS (lumpectomy without radiotherapy), MT+RT (mastectomy following radiotherapy) and MT (mastectomy without radiotherapy). LNR was calculated by dividing the number of positive LNs by the total number of removed LNs. We first investigated the prognostic value of LNR as a continuous variable. Patients were also divided into 3 LNR risk groups (low risk: ≤0.20, intermediate risk: 0.21–0.65, and high risk: >0.65). The chosen LNR cut-off ratios were based on previously published studies [19].

### Statistical analyses

Disease specific survival (DSS) was used as the primary endpoint. Survival was measured from the date of diagnosis to the date of death, the date last known to be alive, or 31 December 2011, when SEER ended the collection of data. For DSS, data for patients who died from causes other than breast cancer were censored at the time of death. To determine the effect of LNR on DSS, we performed a univariate survival analysis using the Kaplan-Meier method and log-rank test. A multivariate analysis of DSS was performed using the Cox proportional-hazards model with both forward and backward stepwise inclusion of factors, with an inclusion criterion of P ≤ 0.01 and an exclusion criterion of P > 0.01. Variables evaluated were age, tumor size, LNR (as both continuous variable and categorical variable), ER/PR status, histologic grade, race and marital status. All tests were 2-sided, and a p-value of < 0.05 was considered statistically significant. All analyses were performed using SAS software (version 9.3; SAS Institute Inc., Cary, NC).

## Results

A total of 53,109 female patients with T1-T2 tumor and one or two positive axillary lymph nodes were identified from the SEER database. The median age at diagnosis was 57 years (range, 18–103 years), and the median follow-up time was 94 months (range, 0–167 months). Baseline characteristics of the patients receiving BCS+RT, BCS, MT+RT and MT were listed in Table 1. To examine the validity of the LNR categories we used in the population of the current study, we did an analysis on DSS of different LNR categories in the selected cohort of 1–2 positive nodes. The 10-year DSS rates were 86%, 85% and 82% for the LNR low, intermediate-, and high-risk groups, with P value less than 0.001. This suggested that the cut-off value currently used can effectively stratifiy breast cancer patients with 1–2 positive nodes into different risk group.

**Table 1:** Baseline Characteristics.

	BCS+RT	BCS	MT	MT+RT	Total
No. of patients	20602	6811	21032	4664	53109
Median age (Range)	57 (21–100)	57 (22–103)	60 (22–100)	53 (23–97)	57 (21–103)
Year of diagnosis					
1998–2002	7628 (37.03%)	2350 (34.50%)	7917 (37.64%)	1846 (39.58%)	19741 (37.17%)
2002–2006	12974 (62.97%)	4461 (65.50%)	13115 (62.36%)	2818 (60.42%)	33368 (62.83%)
T1	13805 (67.01%)	4240 (62.25%)	10747 (51.10%)	1688 (36.19%)	30480 (57.39%)
T2	6797 (32.99%)	2571 (37.75%)	10285 (48.90%)	2976 (63.81%)	22629 (42.61%)
Grade I-II	12634 (61.32%)	3892 (57.14%)	11793 (56.07%)	2256 (48.37%)	30575 (57.57%)
Grade III-IV*	7297 (35.42%)	2622 (38.50%)	8014 (38.10%)	2144 (45.97%)	20077 (37.80%)
ER positive	15785 (76.62%)	4585 (67.32%)	14755 (70.16%)	3220 (69.04%)	38345 (72.20%)
PR positive	13462 (65.34%)	3798 (55.76%)	12188 (57.95%)	2702 (57.93%)	32150 (60.54%)
One positive lymph node	15141 (73.49%)	4820 (70.77%)	14462 (68.76%)	2627 (56.33%)	37050 (69.76%)
Two positive lymph nodes	5461 (26.51%)	1991 (29.23%)	6570 (31.24%)	2037 (43.67%)	16059 (30.24%)
No. of lymph nodes removed					
Median	11 (1–57)	10 (1–48)	12 (1–90)	12 (1–62)	11 (1–90)
1–3	3446 (16.73%)	1265 (18.57%)	2095 (9.96%)	445 (9.54%)	7251 (13.65%)
4–6	2637 (12.80%)	817 (12.00%)	2323 (11.05%)	566 (12.14%)	6343 (11.94%)
6–9	2932 (14.23%)	1001 (14.70%)	3280 (15.60%)	709 (15.20%)	7922 (14.92%)
≥ 10	11587 (56.24%)	3728 (54.73%)	13334 (63.40%)	2944 (63.12%)	31593 (59.49%)
Lymph node ratio					
≤ 0.02	15084 (73.22%)	4780 (70.18%)	16743 (79.61%)	3565 (76.44%)	40172 (75.64%)
>0.2 and ≤0.65	4068 (19.75%)	1367 (20.07%)	3373 (16.04%)	840 (18.01%)	9648 (18.17%)
>0.65	1450 (7.04%)	664 (9.75%)	916 (4.36%)	259 (5.55%)	3289 (6.19%)

*Abbreviations*: BCS = breast-conserving surgery; MT = mastectomy; RT = radiotherapy.

* Grade IV stands for undifferentiated, anaplastic, or not differentiated in the SEER coding system.

**Fig 1:**
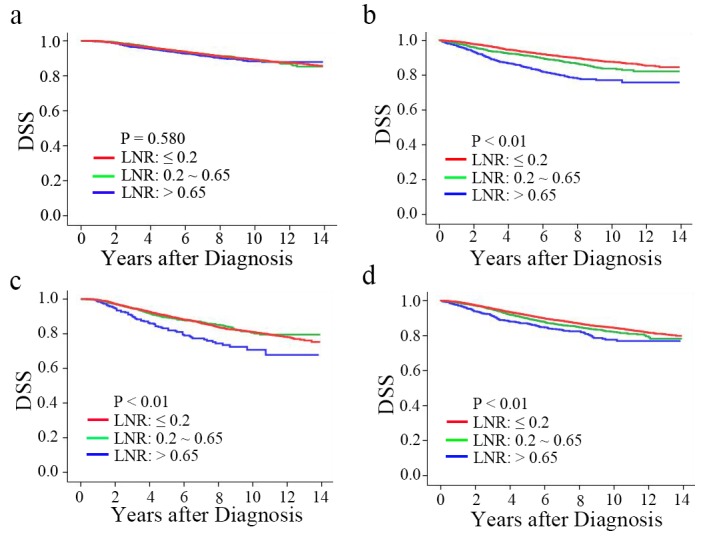
a-d. Kaplan-Meier DSS estimates of breast cancer patients with T1-T2 tumor and 1–2 nodes treated with BCS+RT, BCS, MT, or MT+RT: (a) Disease-specific survival according to LNR in patients receiving BCS+RT; (b) Disease-specific survival according to LNR in patients receiving BCS alone; (c) Disease-specific survival according to LNR in patients receiving MT+RT; (d) Disease-specific survival according to LNR in patients receiving MT alone.

In the subgroup of 20,602 patients who underwent BCS+RT, the continuous variable of LNR did not show significant correlation with DSS in the multivariate model. In the multivariate analyses, age, T staging, ER/PR status, histological grade, race and marital status were initially included in the model as potential risk factors. Factors that were not selected by the stepwise procedure were subsequently omitted. When classifying the patients into LNR low-, intermediate-, and high-risk groups based on previously established cut-offs, there was no difference in DSS between these LNR risk groups (Fig 1A, p = 0.58). The 10-year DSS rates were 89%, 89%, and 88%, respectively. In the multivariate analysis, the categorized variable of LNR was not significantly associated with DSS (Table 2).

**Table 2:** Multivariate analysis of disease-specific survival (DSS) for lymph node ratio (LNR) in patients who underwent BCS+RT, BCS, MT and MT+RT.

Variable	Reference	BCS+RT (n = 20602)			BCS (n = 6811)			MT (n = 21032)			MT+RT (n = 4664)		
		HR	95% CI	P	HR	95% CI	P	HR	95% CI	P	HR	95% CI	P
Age at diagnosis													
** ** 40–59 yr (s)	Age 20–39 yr (s)	0.71	0.65–0.78	<0.01	0.75	0.65–0.86	<0.01	0.66	0.61–0.71	<0.01	0.81	0.71–0.93	<0.01
** ** ≥60 yr (s)	Age 20–39 yr (s)	-*	-	-	-	-	-	-	-	-	-	-	-
Race													
** ** Black	White	1.32	1.15–1.50	<0.01	1.59	1.33–1.91	<0.01	1.43	1.29–1.60	<0.01	-	-	-
** ** Others	White	-	-	-	-	-	-	-	-	-	-	-	-
Married status	Not married, unknown	0.85	0.78–0.93	<0.01	-	-	-	0.82	0.76–0.89	<0.01	0.69	0.60–0.79	<0.01
T2 stage	T1 (size ≤ 2cm)	2.05	1.87–2.25	<0.01	1.88	1.63–2.17	<0.01	1.81	1.67–1.95	<0.01	1.38	1.18–1.62	<0.01
Hormone receptor													
** ** ER+,PR+	ER+/-, -/+,unknown	0.61	0.55–0.69	<0.01	0.63	0.54–0.73	<0.01	0.76	0.69–0.83	<0.01	0.62	0.54–0.72	<0.01
** ** ER-,PR-	ER+/-, -/+,unknown	1.23	1.08–1.39	<0.01	-	-	-	1.62	1.47–1.80	<0.01	-	-	-
Histologic grade III-IV	Grade I-II, unknown	1.95	1.76–2.16	<0.01	1.84	1.59–2.14	<0.01	1.62	1.50–1.76	<0.01	1.71	1.47–1.99	<0.01
IDC	ILC	-	-	-	-	-	-	-	-	-	-	-	-
LNR													
** ** >0.2 and ≤0.65	≤ 0.02	-	-	-	1.49	1.25–1.76	<0.01	1.27	1.15–1.40	<0.01	-	-	-
** ** >0.65	≤0.0 2	-	-	-	2.43	2.00–2.95	<0.01	1.61	1.36–1.90	<0.01	1.74	1.34–2.25	<0.01

*Abbreviations*: BCS = breast-conserving surgery; MT = mastectomy; RT = radiotherapy.

* Variables eliminated by stepwise method.

Additional analyses were conducted by applying the LNR risk categories to the remaining subgroups, including patients who received BCS (n = 6,811), MT (n = 21,032) and MT+RT (n = 4,664). In each of these subgroups, the Kaplan-Meier curve for DSS was significantly separated between different LNR risk groups (Fig 1B, 1C and 1D). The 10-year DSS rates of each treatment group stratified by LNR categories were listed in Table 3. Multivariate analyses also demonstrated the significant correlation of LNR with DSS in the above subgroups (Table 2).

**Table 3:** Disease-specific survival (DSS) rates in each treatment group stratified by LNR categories.

	BCS+RT	BCS	MT+RT	MT
**10-year DSS rate**				
LNR low risk	0.89 (0.88–0.90)	0.88 (0.86–0.90)	0.81 (0.79–0.83)	0.84 (0.83–0.85)
LNR intermediate risk	0.89 (0.87–0.91)	0.84 (0.82–0.86)	0.81 (0.77–0.85)	0.82 (0.80–0.84)
** ** LNR high risk	0.88 (0.86–0.90)	0.77 (0.74–0.81)	0.71 (0.63–0.79)	0.78 (0.74–0.82)

*Abbreviations*: BCS = breast-conserving surgery; MT = mastectomy; RT = radiotherapy.

## Discussion

The absolute number of positive axillary LNs is traditionally accepted as an important prognostic factor in breast cancer patients, reflected in the current TNM classification [25]. In the past decade, several studies have highlighted the prognostic value of LNR. Using a bootstrap sampling method, Vinh-Hung et al [19] identified cutoff values for LNR which classified patients into low- (≤0.20), intermediate- (0.21–0.65), and high-risk (>0.65) LNR groups. They concluded that these LNR cutoff values predict breast cancer prognosis more accurately than pN categories and could be considered as an alternative to pN staging.

Even in some low-risk breast cancer patients, the prognostic value of LNR has been demonstrated [26]. Kim et al analyzed 3,477 patients with T1-T2 and pN1 breast cancer, and verified the clinical significance of LNR categorized by cutoff values similar to the Vinh-Hung et al’s study. Another cohort study included 309,216 cases from the National Cancer Database (NCDB) with tumors up to 5 cm and one or two positive nodes [27]. The authors concluded that LNR is an independent predictor of OS in this low-risk population and that the number of lymph nodes examined may influence the accuracy of prognostic stratification. However, they did not have data regarding type of surgery and thus were unable to sub-classify patients accordingly.

Although these studies have demonstrated the important role of LNR in node-positive breast cancer patients, the debate on the extent of axillary surgery has continued since the publication of the ACOSOG Z0011 trial results [10]. This clinical trial included patients with T1-T2 tumors and negative clinical node status and one or two positive SLNs. Participants were randomized to undergo ALND or no further axillary surgery. All participants received breast-conserving therapy with radiotherapy. Adjuvant systemic therapy was administrated in more than 90% of the participants. After a median follow-up of 6.3 years, no difference was shown for locoregional control and OS between the two treatment groups. Based on these results, the current St. Gallen Consensus guidelines recommended that axillary dissection could be omitted in the context of breast-conserving surgery and radiotherapy for patients with one or two positive sentinel nodes. The panel was divided regarding whether this practice could be applied to mastectomy following radiotherapy, although was almost unanimous in the need for axillary dissection if no radiotherapy was delivered [28].

One goal of our study was to investigate whether the Z0011 trial results could apply to a SEER population. An analysis was conducted on a comparable subgroup of 20,602 patients with T1-T2 tumors,and one or two positive lymph nodes who had been treated with BCS+RT. LNR was not found to be significantly associated with DSS in both the univariate and multivariate model. These results demonstrated that the number of lymph nodes dissected did not influence DSS in this particular low-risk population. We use DSS as the primary endpoint for the consideration that it specifically describes the mortality caused by breast cancer. Since this analysis is based on a large, population-based cohort with limited follow-up duration, OS rate may largely be confounded by deaths due to causes other than breast cancer. Therefore, we believe that our results support the concept that axillary node dissection could be omitted in select patients with positive LNs.

Several variables were included in the multivariate analysis, including disease relevant ones (tumor size, hormonal status, grade, LNR, etc.). We also included race and marital status in this analysis, for the reason that these two factors has been shown to influence breast cancer survival in some previous studies [29, 30, 31]. In a SEER based study of the impact of marital status on survival of patients with cancer, after adjusting for demographics, tumor and nodal stage, and use of definitive therapy, breast cancer patients who were married were significantly less likely to die of their disease (HR, 0.80; 95% CI, 0.79 to 0.81;P < .001). For breast cancer patients, the survival benefit associated with marriage was larger than the published survival benefit of chemotherapy [31]. HER-2 status was not included in this analysis because it was not available in the SEER database.

Another subgroup analysis was conducted on 4,664 patients treated with MT+RT, 6,811 treated with BCS and 21,031 with MT. LNR was an independent predictor of DSS in each of these subgroups. These results may support the conclusion that ALND should not be omitted in the above circumstances. Importantly, it should be noted that more patients in the MT and MT+RT group had T2 disease and two positive LNs than the BCS and BCS+RT group. Thus, this assumption needs careful evaluation. As others have shown, adequate axillary surgery not only provides additional information to predict prognosis and guide treatment planning, it may also improve survival [7,32,33]. However, as ALND has been shown to be associated with a significant risk of complications such as lymphedema, arm paresthesia and pain, the potential benefit must be weighed against the morbidity of the procedure [34–36].

We recognize there are limitations with our study. Firstly, this is a retrospective analysis of a population-based database and, therefore, is prone to confounding and bias. Secondly, we do not have data regarding systemic therapy. One of the significant characteristics of the Z0011 trial population is that more than 90% of them received adjuvant systemic therapy. However, given current practices, the majority of breast cancer patients with a positive LN will be treated with systemic therapy. In an analysis of the NCDB database with similar inclusion criteria, only 17% of the patients did not receive either systemic chemotherapy or endocrine therapy [27]. Another limitation is that the LNR categories used in this study are based on the results of Vinh-Hung et al., which were generated based upon a patient population different from the population in the current study. Furthermore, information on locoregional recurrences or distant metastases was not available in the SEER database. We therefore were unable to determine the prognostic value of LNR in predicting disease-free survival. However, our findings on the influence of the number of resected LNs on DSS may have clinical implications for treating node-positive breast cancer patients.

In summary, our results *add evidence to the concept* that axillary dissection could be omitted in patients with one or two positive nodes following breast-conserving surgery and whole breast radiation. Our results also provide rationale for prospective trials to determine the potential benefit of ALND in patients undergoing mastectomy and those not receiving radiotherapy.
